# Dystrophin Expressing Chimeric (DEC) Cell Therapy for Duchenne Muscular Dystrophy: A First-in-Human Study with Minimum 6 Months Follow-up

**DOI:** 10.1007/s12015-023-10530-4

**Published:** 2023-03-31

**Authors:** Ahlke Heydemann, Grzegorz Bieganski, Jacek Wachowiak, Jarosław Czarnota, Adam Niezgoda, Krzysztof Siemionow, Anna Ziemiecka, Maria H. Sikorska, Katarzyna Bozyk, Stefan G. Tullius, Maria Siemionow

**Affiliations:** 1 Department of Physiology and Biophysics, University of Illinois at Chicago, Chicago, IL USA; 2 Center for Cardiovascular Research, University of Illinois at Chicago, Chicago, IL USA; 3 Department of Infectious Diseases and Child Neurology, Poznan University of Medical Sciences, Poznan, Poland; 4 Department of Pediatric Oncology, Hematology and Transplantology, Poznan University of Medical Sciences, Poznan, Poland; 5 Hospital MedPolonia, Poznan, Poland; 6 Department of Neurology, Poznan University of Medical Sciences, Poznan, Poland; 7 Dystrogen Therapeutics Corp., Chicago, IL USA; 8 Department of Orthopaedics, University of Illinois at Chicago, Chicago, IL USA; 9 Division of Transplant Surgery and Transplant Surgery Research Laboratory, Department of Surgery, Brigham and Women’s Hospital, Harvard Medical School, Boston, MA USA; 10 Department of Traumatology Orthopedics and Hand Surgery, Poznan University of Medical Sciences, Poznan, Poland

**Keywords:** Stem Cell Therapy, Duchenne Muscular Dystrophy, Dystrophin Expressing Chimeric (DEC) Cell, Safety, Electromyography (EMG)

## Abstract

Duchenne Muscular Dystrophy (DMD) is a X-linked progressive lethal muscle wasting disease for which there is no cure. We present first-in-human study assessing safety and efficacy of novel Dystrophin Expressing Chimeric (DEC) cell therapy created by fusion of patient myoblasts with myoblasts of normal donor origin. We report here on safety and functional outcomes of the first 3 DMD patients**.** No study related adverse events (AE) and no serious adverse events (SAE) were observed up to 14 months after systemic-intraosseous administration of DEC01. Ambulatory patients showed improvements in functional tests (6-Minute Walk Test (6MWT), North Star Ambulatory Assessment (NSAA)) and both, ambulatory and non-ambulatory in PUL, strength and fatigue resistance which correlated with improvement of Electromyography (EMG) parameters. DEC01 therapy does not require immunosuppression, involves no risks of off target mutations, is not dependent upon the causative mutation and is therefore a universal therapy that does not use viral vectors and therefore can be readministered, if needed. This study was approved by the Bioethics Committee (approval No. 46/2019).

**Graphical Abstract:**

Mechanism of action of the Dystrophin Expressing Chimeric Cell (DEC) cells created via ex vivo fusion of human myoblast from normal and DMD-affected donors. Following systemic-intraosseous administration, DEC engraft and fuse with the myoblasts of DMD patients, deliver dystrophin and improve muscle strength and function. (Created with BioRender.com)

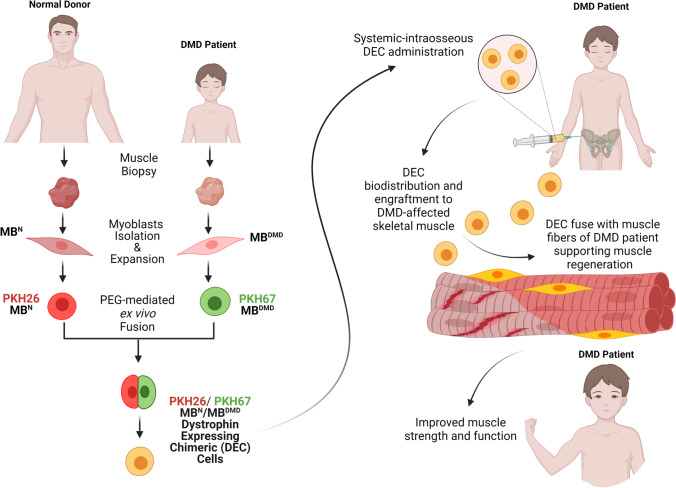

## Introduction

Duchenne Muscular Dystrophy (DMD) is a progressive muscle wasting disease that affects all muscle groups: cardiac, limb skeletal muscles, diaphragm and even smooth muscles. All DMD patients display severe skeletal muscle wasting. Most patients (79.9%) die of cardiopulmonary complications [1–3]*.* Most patients receive corticosteroids at young age (4–8 years) [4], beta-blockers or angiotensin receptor blockers are often given prophylactically around the age of 10 years [5] and nighttime assisted ventilation is added during adolescence (15–18 years) [6]*.* These strategies have improved the lives of patients, however, none of the supportive therapies either improved or halted progression of the disease. Thus, novel therapeutic approaches are required. These therapies may be stand alone or may be administered in combination with the current standards of care, or other new therapies.

Transplantation of muscle stem cells has long been hypothesized to be effective [7–9]*.* Challenges with this approach included detrimental immune responses to the transplanted donor cells. Multiple types of cells to be transplanted, multiple sources of the cells, and various ex vivo manipulations have been investigated [10–21]. Using the patient’s own cells holds the potential to circumvent the detrimental immune response. However, these autologous cells would require ex vivo gene editing to establish dystrophin expression [22–28]. These manipulations may, in turn affect the cells stemness and their proliferation ability. Alternatively, an allogenic HLA-matched donor could be identified. While identifying a matching donor represents a challenge, the recipient may still require supportive immunosuppressive therapy to prevent cell rejection.

A second large issue with cell transplantation strategies has been that the cells must be delivered globally so that all of the affected muscles will receive the dystrophin expressing cells. Global delivery of transplanted cells has until now been attempted with intracardiac, intravenous, or even intraarterial injections [12, 14, 29–32]. However, an effective distribution has not been uniformly achieved [9]. Historically, myoblast-based therapies have been delivered via local-intramuscular injection, thus allowing for a local effect within the injected muscle, however no evidence of global effect have been reported [7–9]. In contrast, delivery of cell-based therapies via intraosseous injections demonstrated systemic effects and have been used with success in both the preclinical and clinical transplantation studies of the umbilical cord blood cells, bone marrow cells, or MSC in both, the adult as well as pediatric population [33–39]*.* Seeding the iliac crest bone marrow cavity with muscle stem cells has not – to our knowledge – been previously reported. The preclinical experiments have identified that intraosseous injections are effective in global delivery of the transplanted cells [33, 35, 37, 40–44].

Murine preclinical trials have demonstrated that the combination of these two techniques significantly improved the muscular dystrophy phenotypes [37, 40–42]. The chimeric cells injected via the intraosseous route: populated the cardiac and all of the skeletal muscles assessed [40], differentiated into muscle cells in these tissues [41], were not identified to be present in non-target tissues [42], did not require immunosuppressives [36, 37, 40, 45], significantly improved function of skeletal muscle [40], diaphragm [40, 41] and cardiac [37, 40] tissues and maintained all of these benefits for at least 180 days [41, 42] while safety was confirmed [42].

Here, we introduce to our knowledge for the first time, the successful use of these two novel techniques in DMD patients. We report on the safety and primary efficacy of a single intraosseous delivery of Dystrophin Expressing Chimeric cells in the first three patients enrolled in this first-in-human Pilot study.

## Materials and methods

### Study design and participants

This single-site, open-label, Pilot study was initiated on August 26, 2021, to assess safety and efficacy of a systemic-intraosseous administration of a single dose of DEC01 therapy. Study protocol and all procedures employed in the study were approved by the Bioethics Committee at the Regional Medical Council in Poznan, Poland (approval no. 46/2019). The study was conducted in accordance with the Good Clinical Practice (GCP) guidelines and the Declaration of Helsinki. Written informed consent for the participation in the study and for a muscle tissue biopsy was received from the donors, participants' parents or legal guardians and from the participants over the age of 13 years.

**Fig. 1:**
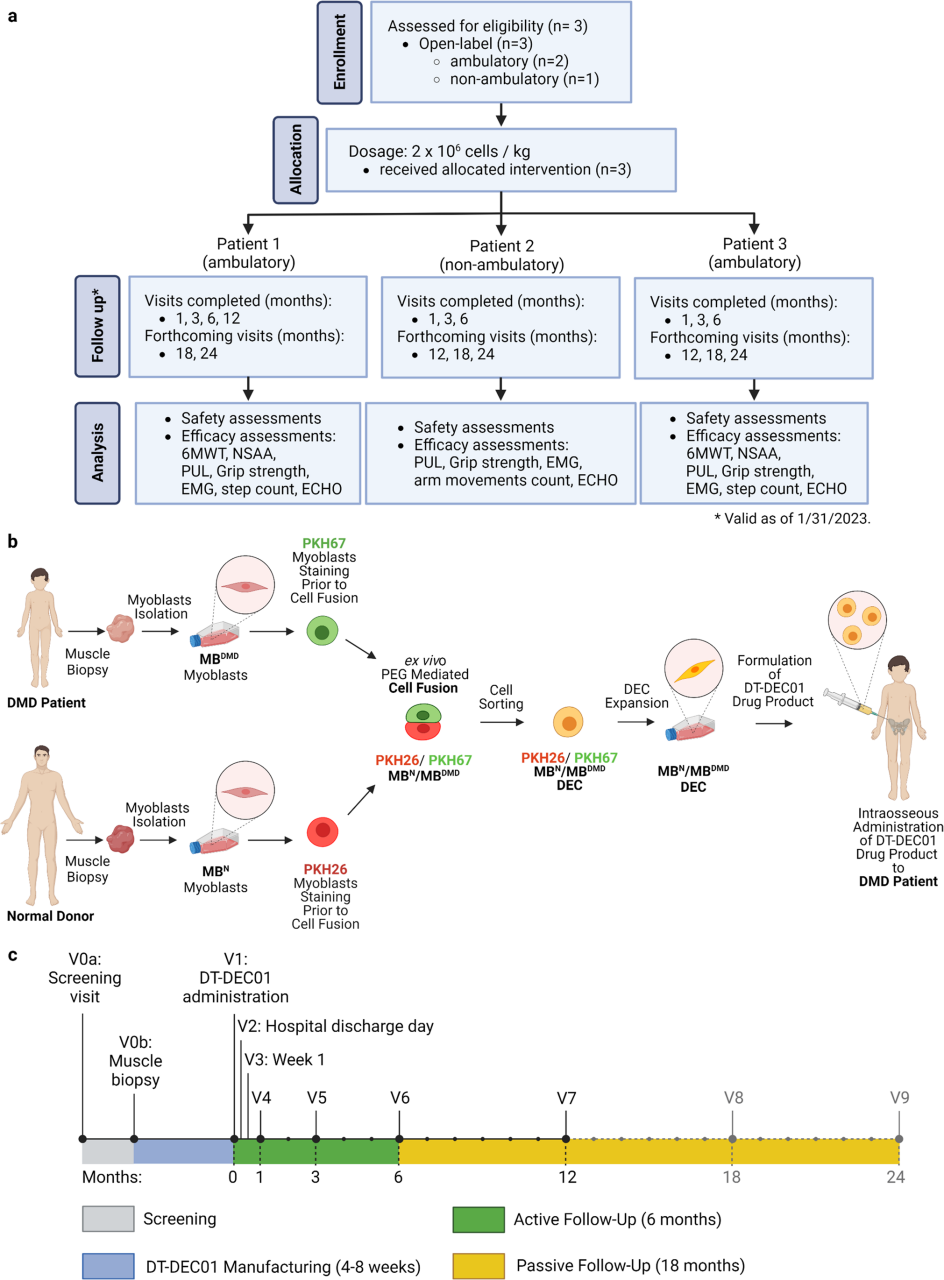
Outline of the first-in-human Pilot study assessing safety and efficacy of systemic-intraosseous administration of DEC01 therapy in DMD patients (**a**) CONSORT diagram of participant inclusion through the study: Enrollment: The ongoing First-in-human pilot study is a single-center, nonrandomized open-label interventional study (Bioethics Committee approval no. 46/2019) performed in male DMD patients aged 5–18. Subjects were screened during the course of the study from a single study site. Allocation: Three patients (both ambulatory (Patient 1 and Patient 3) and non-ambulatory (Patient 2) that met the inclusion criteria were allocated to the 2 × 10^6^ cells/kg body weight dose of intervention. Follow-up: The total study duration is 24 months. As of 1/31/2023 all three patients completed the follow-up visits at 1-, 3- and 6-months after intraosseous DEC01 administration and one patient (Patient 1) completed the 12-month visit. Analysis: For all three patients, the outcomes of safety (assessed by incidence of AEs and SAEs and presence of anti-HLA antibodies) and efficacy (measured by functional assessments adjusted to the stage of the disease: ambulatory patients: 6MWT, NSAA, PUL, grip strength, EMG, step count, ECHO; non-ambulatory patients: PUL, grip strength, EMG, arm movements count, ECHO) were collected at subsequent follow-up visits and analyzed. (**b**) Detailed manufacturing of DT-DEC01, starting with muscle biopsies from DMD patient and normal donor, followed by myoblasts isolation, primary culture, expansion, PKH staining and PEG fusion followed by DEC sorting, expansion, DEC01 product formulation and administration to DMD patient. (**c)** Patients visits schedule: V0a – Screening visit, V0b – skeletal muscle biopsy of DMD patient and normal donor, V1 –intraosseous DEC01 administration. Active follow-up period of 6-months: Visit 2—Hospital Discharge Day; V3 – Week 1; V4 – Month 1; V5 – Month 3; V6—Month 6; passive follow-up visits: V7 – Month 12, V8 – Month 18, V9 – Month 24. Abbreviations**:** DEC—Dystrophin Expressing Chimeric (cells), MB^DMD^—myoblasts derived from the DMD patient, MB^N^ – myoblasts derived from the normal donor. Figure created with BioRender.com

Based on the inclusion and exclusion criteria, the enrolled participants included three male patients of age 6–15 years old suffering from genetically confirmed DMD, irrespective of the existing mutation type and the ambulatory status (Table 1A). Patients and their respective donors underwent the screening visit for verification of their medical history, physical examination and serological status. Since the primary aim was to determine safety, both the ambulatory (n = 2) and non-ambulatory (n = 1) boys were enrolled to the study. Participants were sequentially assigned to undergo muscle tissue biopsy followed by dosing of 2 × 10^6^ cells/kg of DEC01 therapy via systemic-intraosseous administration (Table 1B). This study design is outlined in Fig. 1.

**Table 1:** Characteristics of DMD patients at baseline and specifications of a single dose of DEC01 (2×10^6^ cells per kg) therapy

A. DMD patients’ characteristics at the baseline								
Patient ID	DMD mutation	Age at DMD diagnosis (years)	Age at enrollment (years)	Height (cm)	Weight (kg)	BMI	Duration on steroids before treatment (years)	Functional status
Patient 1	Exon 3-12 deletion	6	6	118	21	15.1	0.5	ambulatory
Patient 2	Exon 48-50 deletion	4	15	147	65	30.0	11.0	wheelchair from the age of 11 years and 10 months
Patient 3	Nonsense mutation	4	6	107	16	14.2	2.0	ambulatory
Average	NA	4.7 ± 0.7	9 ± 3	124.0 ± 11.9	34.0 ± 15.6	19.8 ± 5.1	4.5 ± 3.3	NA
B. Specifications of a single dose of DEC01 (2×10^6^ cells per kg) therapy								
Patient ID	Patient biopsy site (muscle)	Donor biopsy site (muscle)	DEC01 dose (number of cells)		DEC01 administration (date)	Post-transplant follow-up (days / months)*		Follow-up visits completed (months)*
Patient 1	vastus lateralis	vastus lateralis	40.2 × 10^6^		11/26/2021	431 / 14 months		1, 3, 6, 12
Patient 2	biceps brachii	quadriceps femoris	130.0 × 10^6^		02/09/2022	356 / 12 months		1, 3, 6
Patient 3	biceps brachii	quadriceps femoris	32.0 × 10^6^		03/18/2022	319 / 10 months		1, 3, 6

*Valid as of 1/31/2023. Average values are reported as mean ± SEM

### Assessment of the donor-recipient HLA matching

Blood samples were collected from DMD patients and normal tissue donors for the low-resolution typing of HLA class I (A, B and C) and class II (DQB1 and DRB1) antigens (Table 2A) using PCR-rSSO (polymerase chain reaction – reverse sequence-specific oligonucleotide) / PCR-SSP (polymerase chain reaction – sequence-specific primer) methods on Luminex platform and in accordance with Marsh et. al., 2010 [46]. There was no inclusion criterion for HLA match in the study protocol, however HLA testing was essential to determine possible Donor-Recipient incompatibility if the patient showed donor specific antibodies (DSA) at the screening.

**Table 2:** Baseline characteristics of DMD patients and the normal donors by HLA typing and assessment of chimerism by the PCR-rSSOP and STR-PCR analysis at 6 months after systemic – intraosseous DEC01 administration

A. HLA Typing for Class I (A, B and C) and Class II (DRB1 and DQB1) alleles specific for DMD patients (Patient 1, 2 and 3) and the normal donors (Donor 1, 2 and 3)																		
Patient and Donor ID	Patient/Donor Relation	Age (years)		HLA Alleles								HLA match		Anti-HLA antibodies				
				A		B	C		DRB1		DQB1			At baseline		At months 1,3,6		
Patient 1	son	6		11, 24		27, 44	5, 12		8, 12		3 (DQ7), 6	5/10		negative		negative		
Donor 1	father	38		2, 24		44, 51	5, 16		12, 13		3 (DQ7), -			-		**-**		
Patient 2	son	15		3, 11		7, 35	3,7		8,15		4,6	5/10		negative		negative		
Donor 2	father	54		3, 25		7, 18	7, 12		4, 15		3,6			**-**		**-**		
Patient 3	son	6		11, 25		18, 51	4, 12		15, -		5, 6	5/10		negative		negative		
Donor 3	father	43		2, 25		15, 18	3, 12		13, 15		6, -			**-**		**-**		
B. PCR-rSSOP analysis of DEC01 for the presence of the parent cell specific HLA alleles from the DMD patients and the normal donors																		
Patient ID	Cells ID		Cells passage	HLA Alleles														
				A			B			C		DRB1		DQB1				
Patient 1	MB^N^		3	Not assessed*														
	MB^DMD^		2															
	MB^N^/MB^DMD^ DEC01		5															
Patient 2	MB^N^		2	3, 25			7, 18			7, 12		4, 15		3, 6				
	MB^DMD^		2	3, 11			7, 35			3, 7		8, 15		4, 6				
	MB^N^ /MB^DMD^ DEC01		5	3, 11, 25			7, 18, 35			3, 7, 12		4, 8, 15		3, 4, 6				
Patient 3	MB^N^		2	2, 25			15, 18			3, 12		13, 15		6, -				
	MB^DMD^		2	11, 25			18, 51			4, 12		15, -		5, 6				
	MB^N^/MB^DMD^ DEC01		7	2, 11, 25			15, 18, 51			3, 4, 12		13, 15, -		5, 6				
C. STR-PCR analysis of DEC01 for the presence of parent cells specific loci from the DMD patients and the normal donors																		
Patient ID	Cells ID	Cells passage	STR Loci															
			Penta E	D18S51	D21S11	TH01	D3S1358	FGA	TPOX	D8S1179	vWA	Amelogenin	Penta D	CSF1PO	D16S53	D7S820	D13S317	D5S818
Patient 1	MB^N^	3	Not assessed*															
	MB^DMD^	2																
	MB^N^/MB^DMD^ DEC01	5																
Patient 2	MB^N^	2	7, 10	15, 17	31.2, -	9, 9.3	16, 17	19, 21	8, -	10, 14	16, 18	X, Y	9, -	12, -	12, 13	8, 10	11, 12	11, 13
	MB^DMD^	2	7, 10	17, -	29, 31.2	9, -	16, 17	19, 24	8, -	10, 14	16, -	X, Y	9, 13	11, 12	11, 12	9, 10	12, 14	13, -
	MB^N^ /MB^DMD^ DEC01	5	**7,** **10**	**15,** **17**	**29,** **31.2**	**9,** **9.3**	**16,** **17**	**19,** **21,** **24**	**8,** **-**	**10,** **14**	**16,** **18**	**X,** **Y**	**9,** **13**	**11,** **12**	**11,** **12,** **13**	**8,** **9,** **10**	**11,** **12,** **14**	**11,** **13**
	Donor chimerism per locus (%)		NA **	40%	19%	27%	NA **	23%	NA **	NA **	23%	NA **	37%	21%	22%	28%	34%	28%
	Average (SEM)		27% ± 2.1															
Patient 3	MB^N^	2	7, 13	18, -	30, 31	9.3, -	14, 18	18, 21	8, -	13, 16	18, 19	X, Y	10, 12	10, -	9, 13	7, 11	11, -	12, 13
	MB^DMD^	2	7, -	13, 18	27, 31	9, 9.3	14, 15	21, 24	8, -	13, -	18, -	X, Y	10, 11	10, 12	9, 12	7, 13	11, 12	11, 13
	**MB** ^**N**^ **/MB** ^**DMD**^ **DEC01**	7	**7,** **13**	**13,** **18**	**27,** **30,** **31**	**9,** **9.3**	**14,** **15,** **18**	**18,** **21,** **24**	**8,** **-**	**13,** **16**	**18,** **19**	**X,** **Y**	**10,** **11,** **12**	**10,** **12**	**9,** **12,** **13**	**7,** **11,** **13**	**11,** **12**	**11,** **12,** **13**
	Donor chimerism per locus (%)		15%	10%	NA **	36%	16%	21%	NA **	19%	18%	NA **	19%	25%	19%	20%	18%	31%
	Average (SEM)		21% ± 1.9															

Abbreviations: *MB*^*N*^ myoblasts from normal donor, *MB*^*DMD*^ myoblasts from DMD patient, *MB*^*N*^*/MB*^*DMD*^ *DEC01* chimeric DEC cells derived from the fusion of MB^N^ and MB^DMD^ cells,*PCR-rSSOP* polymerase chain reaction - reverse sequence-specific oligonucleotide probe; STR-PCR polymerase chain reaction short-tandem repeat

* HLA typing of the MB^N^, MB^DMD^ and MB^N^/MB^DMD^ DEC01 was introduced into study protocol before patient 2 enrollment

**Not applicable – the patient and the donor share the same STR loci

### Assessment of the anti-HLA antibodies and Donor Specific Antibodies (DSA)

Patients’ sera were verified for the presence of anti-HLA class I, anti-HLA class II and anti-MICA IgG antibodies (LABScreen Mixed, One Lambda, Luminex platform) at the screening. In the case of a positive result, further analysis was employed to determine the presence of the pre-existing DSA (LABScreen Single Antigen HLA Class I and Class II, One Lambda, Luminex platform). If the DSA were detected in DMD patient, the donor was excluded and another donor for allogeneic myoblast donation was screened.

The anti-HLA testing was repeated during the follow-up visits at 1, 3 and 6 months after systemic – intraosseous DEC01 administration to assess the potential immune response to the DEC01 therapy (Table 2A).

### Manufacturing of the patient specific / personalized DEC01 therapy product

After donor selection, the patient’s (MB^DMD^) and the normal donor’s myoblasts (MB^N^) were obtained from an open muscle biopsy (1-3cm^3^) performed under general anesthesia (DMD patient), and analgosedation, general or local anesthesia (the normal donor) performed at the MedPolonia Hospital. Following biopsy, muscle samples were transferred via medical courier to the Polish Stem Cells Bank (PBKM), where the Dystrogen’s (DT) DT-DEC01 product based on Dystrophin Expressing Chimeric (DEC) cells (further described as DEC01) was manufactured under Good Manufacturing Practice (GMP) conditions according to the manufacturing protocol approved by the Chief Pharmaceutical Inspector of Poland (Fig. 1b). According to the protocol, first, the muscle tissue samples were digested at 37 °C, with 0.454 U/ml collagenase (Nordmark Pharma GmbH), with vigorous agitation for 45 min. The patient’s and donor’s myoblasts were isolated in culture medium: DMEM (HyClone) supplemented with L-Alanyl-L-Glutamine (Biological Industries), ELAREM Ultimate—FDi (PL Bioscience GmbH), Anti-Anti (Gibco-ThermoFisher) and hBFGF (Biotechne). The cells were propagated in animal component-free, GMP grade cell culture medium without antibiotics. When reaching maximum 70% confluency, myoblasts were passaged: cells were harvested using TrypLE TE Select (Gibco-ThermoFisher) and used for fusion between passages 2–4. Prior to fusion, harvested myoblasts after counting and viability assessment with Trypan Blue (Gibco-ThermoFisher) were washed in serum-free media with antibiotics. MB^DMD^ and MB^N^ cells were single-stained with green and red fluorescent membrane dye, respectively (PKH67 and PKH26, Sigma-Aldrich) according to manufacturer’s instructions. Polyethylene glycol (PEG 4000, Merck) with DMSO (WAK-Chemie Medical GmbH) in DMEM was used to fuse the two single-stained cell populations in 1:1 ratio. After fusion, to assure that a pure population of the created DEC cells will be administered to the patient, double-positive DEC cells were selected by the FACS MACSQuant Tyto (Miltenyi Biotec) sorter. The detailed outline of DEC01 product manufacturing is presented in Fig. 1b.

### DEC01 dose and product concentration

Propagation of the DEC cells was continued in vitro after fusion (4–7 passages) until the cell count reached the sufficient number for the formulation of the final DEC01 product. The manufactured dose of the personalized DEC01 therapy for each DMD patient was based on the Primary Investigator order and was calculated based on the patient's body weight.

In the final step of manufacturing process, DEC cells were harvested, filtered (100 µm mesh), suspended in 0.9% sodium chloride solution (Fresenius Kabi) and packaged at the final concentration of 10–20 × 10^6^ cells/ml in the ready-to-use, individually labeled, sterile vial of 2 ml volume (CellSeal) or in its multiplications, depending on the final dose to be administered (Table 1B). Before release to the hospital, DEC01 product was verified for: sterility, endotoxins level and cell viability as part of the standard Quality Control (QC) assessment. Each batch of the personalized DEC01 product was transferred at 2 °C-8°C temperature-controlled ORCA packaging (Intelsius) to the MedPolonia Hospital for administration to the patient (Fig. 1b).

### Assessment of DEC01 product chimerism

For assessment of chimerism in the DEC01 product, myoblasts from normal donor and DMD patient were harvested from the cell culture during DEC01 manufacturing on the day of cell fusion, whereas the created DEC cells were harvested on the day of the final DEC01 product formulation. The cells samples were transferred to the Immunogenetics Laboratory at the Medical University of Warsaw for the polymerase chain reaction—reverse sequence-specific oligonucleotide probe (PCR-rSSOP) and polymerase chain reaction short-tandem repeat (STR-PCR) analysis. DNA isolation was performed using QIAamp DNA Blood Mini kit (QIAGEN) and QIAcube Connect system (QIAGEN) to obtain DNA samples of myoblasts from the donor, the DMD patient and the created DEC cells to be used during analysis of PCR-rSSOP and STR-PCR.

The PCR-rSSOP procedure was performed using LABType rSSO (OneLambda) tests according to the manufacturer’s instructions. Briefly, DNA samples were PCR-amplified using a loci-specific primers, biotinylated to be detected using R-Phycoerythrin-conjugated Streptavidin (SAPE), denatured and rehybridized to complementary DNA probes conjugated to fluorescently coded microspheres. Next, the samples were analyzed using either the LABScan 100 (Luminex 100/200) or LABScan3D (Luminex FLEXMAP 3D) system, identifying each microsphere fluorescent (phycoerythrin) intensity. Each sample of the parent cells was analyzed for the presence of the HLA alleles of class I (A, B and C) and class II (DRB1 and DQB1). The DEC01 product was analyzed for the presence of a combination of HLA alleles specific for normal donor and DMD patient to verify chimeric status of DEC cells. HLA allele names are in accordance with the current HLA nomenclature database [47].

The STR-PCR was performed using PowerPlex 16 HS System (Promega). Briefly, the DNA samples were amplified and subjected to the capillary electrophoresis (Spectrum Compact CE1304, Promega). The raw data was analyzed in ChimerMaker software, according to the manufacturer’s instructions. The samples were analyzed for the following loci: Penta E, D18S51, D21S11, TH01, D3S1358, FGA, TPOX, D8S1179, vWA, Amelogenin, Penta D, CSF1PO, D16S539, D7S820, D13S317 and D5S818. The STR profile of DEC01 product was subjected to analysis for the relative quantities of the STR loci specific for the normal donor and DMD patient in the fused DEC cells. The average donor chimerism (percentage of the donor STR loci in DEC cells) was calculated from the informative (non-shared) loci.

### Systemic-intraosseous administration of the DEC01 therapy

The participants received the personalized DEC01 product via intraosseous delivery route into the bone marrow cavity of the patient’s iliac crest. Each boy was anesthetized and placed in the prone position for aseptic preparation of the operating area of the posterior, upper edge of the iliac crest. Bone marrow was aspirated to create space in the marrow cavity prior to the DEC01 cell suspension transfer from the vial. DEC01 cells were injected to the marrow cavity in one injection site, or multiple sites if the final dose was divided to several vials. After the full dose was injected, the surgery site was pressed by the operator to ensure closure before it was protected with a dressing. The intraosseous administration procedure lasted on average 7 min. Following DEC administration, the patient was hospitalized for 24 h and monitored for any signs of response related to the procedure of systemic-intraosseous DEC01 administration.

### Safety assessment of the DEC01 therapy

Safety was assessed as a primary aim of this Pilot study in terms of the incidence and severity of all treatment-related adverse events (AE) and serious adverse events (SAE). Patients were observed for abnormalities in vital signs, physical examination and laboratory tests, starting from the muscle tissue biopsy through the DEC01 therapy administration to follow-up period of 6 months after administration. Safety evaluation was continued from 6 months of the active follow-up to the 24 months of the passive follow-up for assessment of the SAE. During the first month post-DEC administration, assessment of AE of special interest (AESI) was performed for monitoring of any intra- and post-infusion complications, both the local and systemic.

### Preliminary efficacy assessment of DEC01 therapy by functional tests

Efficacy was assessed by the standard functional tests adjusted to the stage of the disease. The selected tests were performed during the screening visit and compared with the measurements recorded during the follow-up visits at 1, 3 and 6 months after the systemic-intraosseous administration of DEC01 therapy. The electromyography (EMG) assessment was carried out at the baseline and at 3 and 6 months after DEC01 administration. All tests were performed according to the standardized methods, the safety of patients was ensured by providing the appropriate conditions.

For ambulatory patients, 6-Minute Walk Test (6MWT) and timed tests of NorthStar Ambulatory Assessment (NSAA) were assessed. All patients were subjected to the Performance of Upper Limb test (PUL 2.0) and the measurements of the hand grip strength by dynamometer. Electromyography was performed in both, the ambulatory and non-ambulatory patients for the assessment of Motor Units Potentials (MUP) in the selected muscles of upper and lower extremities. Cardiac function was monitored in all patients by echocardiography (ECHO) at all follow-up visits after DEC01 administration. Daily activity was continuously measured with the step or arm movement counter (Vívosmart 4, Garmin).

### The 6-Minute Walk Test (6MWT)

Ambulatory patients were asked to walk 30 m for 6 min at their normal pace. The test was performed indoors on the flat, non-slippery surface. Each end of the walking path was marked with a training cone. Patients were allowed to stand and rest if needed or use their standard gait assisting devices (crutches, cane). The total distance walked was calculated from the number of full 30-m walks and the last partial lap and was expressed in meters.

### NorthStar Ambulatory Assessment (NSAA) timed functions

Ambulatory patients were given instructions to perform NSAA activities testing their overall physical functioning including the two timed tests of standing from the supine position and walking/running 10-m path with maximum, self-selected speed. Time needed to perform these tasks was measured in seconds with a stopwatch. Patients were observed for the compensatory movements, specifically for the presence of the Gowers’s sign during rising from supine.

### Performance of Upper Limb (PUL)

PUL 2.0 test was assessed to evaluate the function of upper limb at high- (shoulder), mid- (elbow) and distal- (wrist and hand) level. The entry task was employed to assess upper limb condition: if the patient was not able to raise a plastic cup with 200 g weight to mouth (score below 3), then the high-level activities of PUL test were not introduced, including the overhead shoulder abduction, shoulder flexion above the head and shoulder flexion with 0.5 – 1 kg weights. The mid-level assessments included raising hands to mouth, moving hands from laps to table, moving weights of 0.1 to 1 kg over the tabletop and lifting loaded cans. Moreover, patients were asked to form a stack of 5 cans and remove the lid from the plastic container. Distal-level tasks included tearing a folded sheet of paper, drawing a line without interruption of the pencil movement, turning on the lamp by pressing with fingers. Additionally, the following activities were assessed at the distal level: supination of the wrist, picking up coins using one hand, moving the finger along the numbers in the diagram and picking up 10 g weight with fingers pinch.

### Assessment of the hand grip strength

The grip strength of both the right and the left hand was quantified by means of a handheld electronic dynamometer (WWEH101, Moga). Patients in seated position were instructed to bend the elbow at 90° and to perform voluntary contraction of each hand applying as much force as possible for 3 consecutive repetitions while holding the dynamometer. The grip strength was recorded in kilograms.

### Electromyography

EMG analysis was performed using needle electrodes connected to the EMG device (Synergy EMG System, Medelec). The needle electrodes were inserted into the selected skeletal muscles, including for the upper extremity: deltoideus and biceps brachii, and for the lower extremity: rectus femoris and gastrocnemius muscle. Patient was instructed to contract muscles by performing specific movements. Electrical activity of muscles was recorded in the form of waves and subsequently analyzed for Motor Unit Potentials (MUP) characteristics, including the time of the MUP duration which positively correlates with the volume of the firing motor units. The average duration of MUP was calculated from an average of n = 10 MUP recordings.

### Echocardiography

Echocardiography (ECHO) was performed with an ultrasound system (Vivid T8, GE Healthcare). The patients in supine position were subjected to the ultrasonographic examination with a transducer probe moved across the chest to assess the real-time heart images. The images of heart structures were obtained to assess cardiac systolic function parameters, including the Ejection Fraction (EF) and Fractional Shortening (FS).

### Step or arm movement assessment by the Garmin counter

Patients were provided with a wristband activity tracker (Vívosmart 4, Garmin). Since the wristbands detected arm movements associated with taking steps, propelling a wheelchair and other daily activities performed with hands, the tracker was employed in both, the ambulatory and non-ambulatory patients. Activity reports were generated for the daily number of steps or arm movements, and the collected data from each month was subjected to statistical analysis. Records from days with breaks in wear time longer than 6 h were excluded from the analysis.

### Statistics

The analysis for statistical significance was performed using GraphPad Prism ver. 9.5.0 software. Data from: the hand grip strength assessment, the duration of the MUP in EMG, and step or arm movement count are shown as mean ± SEM. The normality of the data was verified by the Shapiro–Wilk test. Parametric one-way ANOVA with Tukey's post-hoc test was used for normally distributed data. For data with an asymmetric distribution, the non-parametric Kruskal–Wallis test with Dunn’s multiple comparisons was applied. P values were considered significant below 0.05.

## Results

### Study population and treatment

#### Characteristics of participants enrolled to the first-in-human Pilot study

This Pilot study specifications called for 10 patients to be enrolled. Out of 5 patients screened for eligibility, one patient was excluded due to the lack of matching donor. Currently three DMD patients have been enrolled including two ambulatory (patient 1 & patient 3) and one non-ambulatory patient (patient 2). Baseline characteristics of the patients 1–3, including the demographic data (type of DMD mutation, age at DMD diagnosis, age at enrollment, height, weight and BMI), duration on steroids before treatment and functional status (ambulatory vs. non-ambulatory), are summarized in Table 1A. The summary of DEC01 therapy dose, date of administration and completed visits and post-transplant follow-up time are provided in Table 1B.

The design of this Pilot study is presented in Fig. 1 including a diagram of the study design (Fig. 1a), outline of manufacturing of the DEC01 cell-based therapy (Fig. 1b) and the schedule of the visits (Fig. 1c).

### Clinical Outcomes

#### Confirmation of safety of a single dose of DEC01 therapy up to 14 months after systemic—intraosseous administration

The primary outcome measure for this Pilot study was safety. Therefore, the patients have been carefully and continuously monitored for any and all possible indications of side effects stemming from the one-time/single dose therapy (Fig. 1c). After the first 10–14 months post DEC01 administration none of the patients experienced any adverse events. In detail: participants did not experience Adverse Events of Special Interest (AESI): the patients were free from surgery site inflammation or tenderness, and were free from temperature increases, nausea, or exhaustion. No study related Adverse Events (AEs) or Serious Adverse Events (SAEs) were reported for an average of 369 days (12 months) after DEC01 administration. To objectively measure the lack of an immune response, the anti-HLA antibodies levels were assessed in the serum of all three patients at baseline, before DEC01 administration and at the follow-up visit at 1, 3, 6 and 12-months post-transplant (Fig. 1c). At no time points did any of the patients displayed presence of anti-HLA antibodies (Table 2A), indicating that the transplants were well-tolerated and did not generate immune response. The presence of combination of the HLA specific alleles from both, the donor of normal myoblasts and the DMD patient myoblasts in the DEC cells confirmed creation of the personalized, chimeric DEC01 therapy for DMD patients (Table 2B). Quantitative analysis of short tandem repeats (STR) profiling of the DEC01 product manufactured for patient 2 and patient 3 revealed an average of 27% and 21% of STR loci specific for the donor of normal myoblasts, respectively (Table 2C).

#### Preliminary efficacy outcomes up to 6 months after intraosseous administration of a single dose of DEC01 therapy

**Fig. 2:**
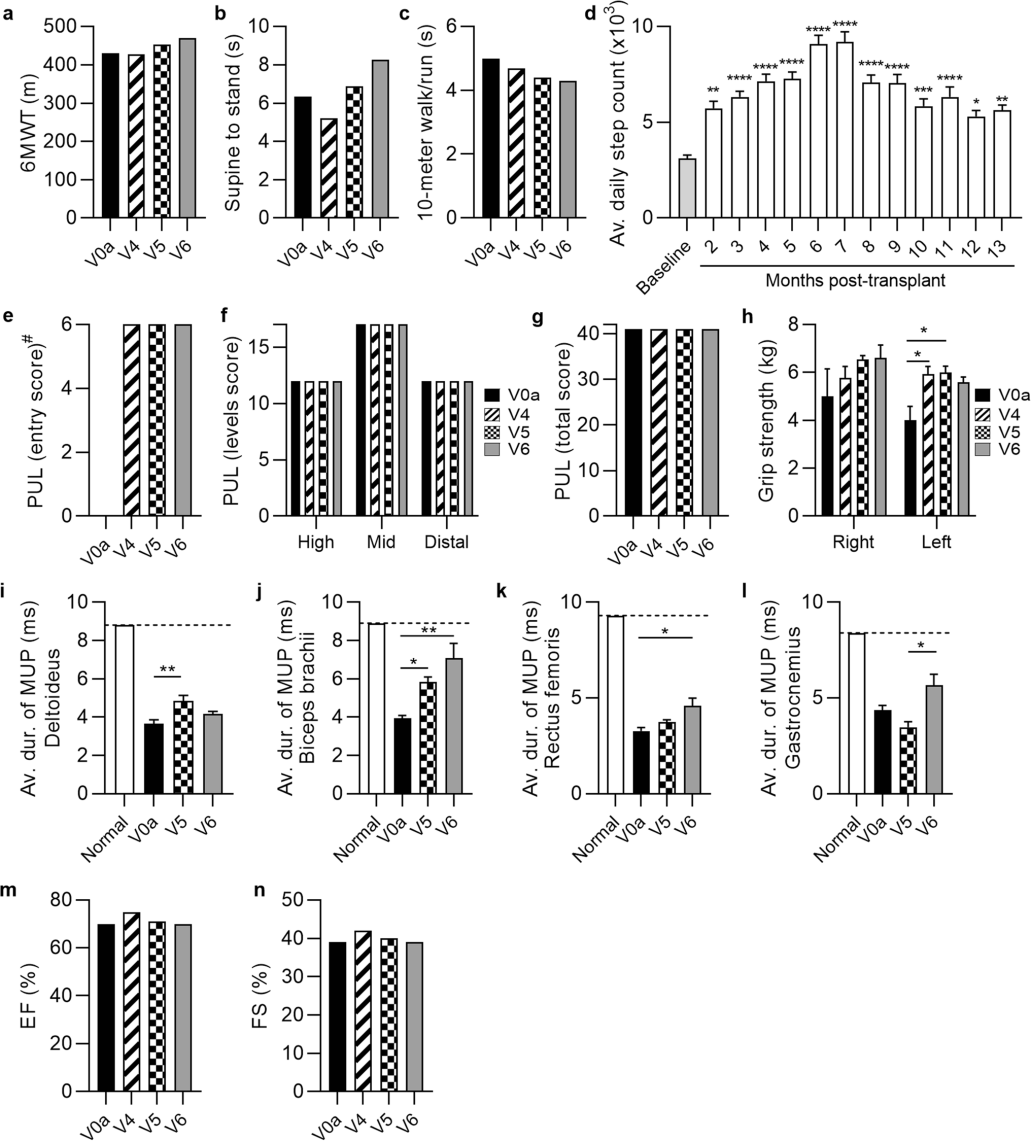
The functional and EMG outcomes assessed in patient 1 up to 6 months after systemic-intraosseous DEC01 administration (**a**) 6MWT revealed 9% increase in the 6 MW distance. (**b**) Time taken to stand from supine was 30% longer. (**c**) Time to walk/run 10 m was 14% shorter. (**d**) Average daily steps count significantly increased over the 13-months follow-up compared to the baseline values. Assessment of PUL (**e–g**) revealed: (**e**) preservation of the entry score (^#^entry task was not performed at V0a visit), (**f**) three domains of upper limb score and (**g**) the total score. (**h**) Assessment of grip strength revealed 32% increase in the right and 40% increase in left hand. EMG assessments revealed increase in the average duration of MUP in: (**i**) deltoideus by 15%, (**j**) biceps brachii by 79%, (**k**) rectus femoris by 40% and (**l**) gastrocnemius by 28%. (**m**) Ejection fraction (EF) and (**n**) fractional shortening (FS) values were maintained at baseline level during 6-months follow-up. Data expressed as mean ± SEM (d, h, i, j, k, l); statistical significance assessed by ANOVA (h) or Kruskal–Wallis test (d, i, j, k, l). * P ≤ 0.05, ** P ≤ 0.01, *** P ≤ 0.001, **** P ≤ 0.0001. Abbreviations: V0a – screening visit, V4 – 1-month, V5—3-months, and V6 – 6-months visits post-transplant

**Fig. 3:**
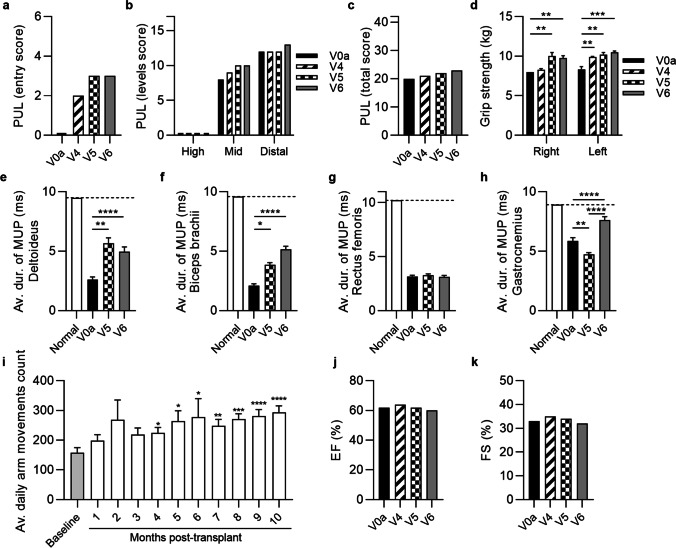
The functional and EMG outcomes assessed in patient 2 up to 6 months after systemic-intraosseous DEC01 administration (**a**) Patient improved in entry task of PUL from 0 (no useful hand activity) to 3 (raising a loaded cup to mouth). (**b**) Assessment of upper limb performance in 3 levels revealed improvements in mid-level (by 25%) and distal-level (by 8%) activities. (**c**) PUL total score improved by 15%. (**d**) Grip strength improved in both right (by 23%) and left (by 27%) hand. The EMG assessments of average duration of the MUP revealed: (**e**) increase by 91% in deltoideus, (**f**) increase by 143% in biceps brachii, (**g**) preservation in rectus femoris and (**h**) increase by 30% in gastrocnemius. (**i**) Average daily count of arm movements increased significantly during follow-up compared to baseline values before transplant. (**j**) Ejection fraction (EF) and (**k**) fractional shortening (FS) values were maintained at baseline level over entire follow-up period of 6-months. Data expressed as mean ± SEM (d, e, f, g, h, i); statistical significance assessed by ANOVA (d, e, f) or Kruskal–Wallis test (g, h, i). * P ≤ 0.05, ** P ≤ 0.01, *** P ≤ 0.001, **** P ≤ 0.0001. Abbreviations: V0a—screening visit, V4 – 1-month, V5 – 3-months, and V6 – 6-months visits post-transplant

**Fig. 4:**
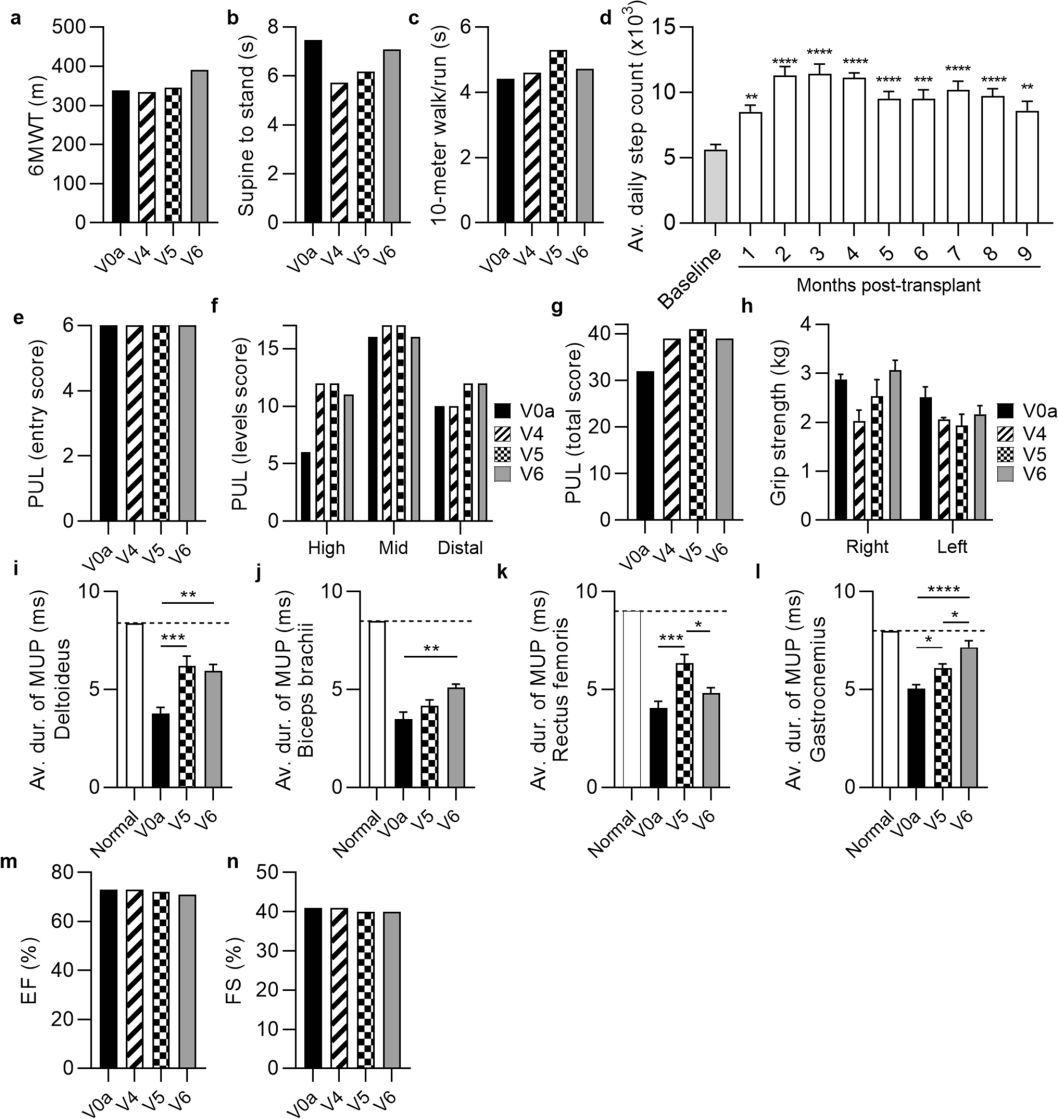
The functional and EMG outcomes assessed in patient 3 up to 6 months after systemic-intraosseous DEC01 administration (**a**) 6MWT revealed 16% increase in the 6 MW distance. (**b**) Time to stand from supine was 5% shorter. (**c**) Time to walk/run 10 m was comparable to baseline. (**d**) Average daily steps count increased during follow-up period. Assessment of PUL (**e–g**) showed (**e**) maintenance of entry score and (**f**) mid-level performance with increase of high- (by 83%), distal-level (by 20%) and (**g**) total (by 22%) PUL score. (**h**) Assessment of grip strength revealed increase in right (by 7%) and deterioration in left (by 12%) hand. EMG assessments revealed improvements in average duration of the MUP compared to baseline: (**i**) deltoideus by 58%, (**J**) biceps brachii by 46%, (**k**) rectus femoris by 19% and (**l**) gastrocnemius by 45%. (**m**) Ejection fraction (EF) and (**n**) fractional shortening (FS) values were maintained at the baseline level over the entire follow-up period of 6-months. Data expressed as mean ± SEM (d, h, i, j, k, l); statistical significance assessed by ANOVA (h, i, j, l) or Kruskal–Wallis test (d, k). * P ≤ 0.05, ** P ≤ 0.01, *** P ≤ 0.001, **** P ≤ 0.0001. Abbreviations: V0a – screening visit, V4 – 1-month, V5—3-months, and V6 – 6-months visits post-transplant

The preliminary efficacy measures were assessed at the screening visit and at 1, 3 and 6 months after systemic – intraosseous administration of a single dose (2 × 10^6^ cells/kg of body weight) of DEC01 therapy and included: for ambulatory patients—the Six-Minute Walk Test (6MWT) and NorthStar Ambulatory Assessment (NSAA), whereas Performance of Upper Limb (PUL 2.0), grip strength measured by dynamometer, and steps or arm movements count measured with Garmin Vívosmart 4 wristband were assessed in both, the ambulatory and the non-ambulatory patients (Fig. 2, 3 and 4, Table 3A). Electromyography (EMG) assessment of the Motor Unit Potentials (MUP) of the selected muscles (Fig. 2, 3 and 4, Table 3B) was performed in both, the ambulatory and non-ambulatory patients. Additionally, cardiac function was assessed by echocardiography (ECHO) (Fig. 2, 3 and 4).

**Table 3:** Summary of efficacy data at 6 months after systemic-intraosseous administration of DEC01 therapy

A. Summary of functional efficacy data by standard functional tests of 6MWT, NSAA, PUL 2.0 and Grip strength at 6 months after systemic-intraosseous administration of DEC01 therapy												
			NSAA		PUL 2.0					Grip strength		Average daily steps or arm movements count
	Parameter	6MWT (m)	Supine to stand (s)	10 m walk (s)	Entry score* (max 6)	High level score (max 12)	Mid level score (max 17)	Distal level score (max 13)	Total score* (max 42)	Right hand (kg)	Left hand (kg)	
Patient 1	Baseline	430	6.35	4.99	6	12	17	12	41	5.0	4.0	3120 ± 163
	Month 6	469	8.27	4.29	6	12	17	12	41	6.6	5.6	9084 ± 459
	Change from baseline at Month 6	39	1.92	–0.70	0	0	0	0	0	1.6	1.6	5964
		9.07%	30.24%	–14.03%	0.00%	0.00%	0.00%	0.00%	0.00%	32.00%	40.00%	191.15%
Patient 2	Baseline	NA**	NA**	NA**	0	0	8	12	20	8.0	8.3	158 ± 17
	Month 6				3	0	10	13	23	9.8	10.5	277 ± 63
	Change from baseline at Month 6				3	0	2	1	3	1.8	2.2	119
					Ability regained	0.00%	25.00%	8.33%	15.00%	22.50%	26.51%	75.32%
Patient 3	Baseline	337	7.48	4.41	6	6	16	10	32	2.9	2.5	5634 ± 363
	Month 6	390	7.08	4.72	6	11	16	12	39	3.1	2.2	9512 ± 692
	Change from baseline at Month 6	53	–0.40	0.31	0	5	0	2	7	0.2	–0.3	3878
		15.73%	–5.35%	7.03%	0.00%	83.33%	0.00%	20.00%	21.88%	6.90%	–12.00%	68.83%
Average change from baseline at Month 6		46	0.76	–0.20	1.00	1.67	0.67	1.00	3.33	1.20	1.17	3230
		12.40%	12.44%	–3.50%	NA	27.78%	8.33%	9.44%	12.29%	20.47%	18.17%	111.77%
B. Summary of electromyographic (EMG) assessment of Motor Unit Potentials (MUP) of the selected muscles at 6 months after systemic-intraosseous administration of DEC01 therapy												
	Parameter		Average duration of MUP (ms)									
			Deltoideus			Biceps brachii		Rectus femoris			Gastrocnemius	
Patient 1	Baseline		3.65 ± 0.21			3.96 ± 0.14		3.27 ± 0.19			4.37 ± 0.25	
	Month 6		4.18 ± 0.12			7.08 ± 0.76		4.59 ± 0.40			5.61 ± 0.58	
	Change from baseline at Month 6		0.53			3.12		1.32			1.24	
			14.52%			78.79%		40.37%			28.38%	
Patient 2	Baseline		2.60 ± 0.23			2.12 ± 0.14		3.16 ± 0.12			5.87 ± 0.27	
	Month 6		4.96 ± 0.40			5.15 ± 0.28		3.11 ± 0.15			7.62 ± 0.28	
	Change from baseline at Month 6		2.36			3.03		–0.05			1.75	
			90.77%			142.92%		–1.58%			29.81%	
Patient 3	Baseline		3.76 ± 0.33			3.49 ± 0.36		4.06 ± 0.34			4.92 ± 0.16	
	Month 6		5.94 ± 0.34			5.10 ± 0.18		4.83 ± 0.27			7.15 ± 0.34	
	Change from baseline at Month 6		2.18			1.61		0.77			2.23	
			57.98%			46.13%		18.97%			45.33%	
	Average change from baseline at Month 6		1.69			2.59		0.68			1.74	
			54.42%			89.28%		19.25%			34.50%	

Average data are presented as mean ± SEM. Abbreviations: *6MWT* Six-Minute Walk Test, *NSAA* NorthStar Ambulatory Assessment, *PUL* Performance of Upper Limb

*The PUL 2.0 entry score is not included in total score

**Parameter not assessed – patient 2 is non-ambulatory. Data are presented as mean ± SEM

The summary of the average outcomes at 6-months post-DEC01 therapy administration for two ambulatory patients revealed improvement in 6MWT, (by an average of 12.40%, 46 m). The average NSAA time of supine to standing maneuver was 0.76 s longer, indicating that on average the 2 ambulatory patients performed less well on this test than at baseline. For the other NSAA timed function – time needed to walk/run 10 m—the average was 0.2 s shorter, indicating that on average the patients performed better than baseline, (Fig. 2, 3 and 4; Table 3A).

Moreover, the outcomes of the PUL 2.0 test in all three patients (both the ambulatory and non-ambulatory) demonstrated an improvement by an average of 12.29%, (3 points) as well as increased grip strength in both, the right hand (by an average of 20.47%, 1.2 kg) and left hand (by an average of 18.17%, 1.2 kg), (Fig. 2, 3 and 4; Table 3A). The EMG assessment revealed an increase of average duration of the MUP in the selected skeletal muscles of: deltoideus (by an average of 54.42%, 1.69 ms), biceps brachii (by an average of 89.28%, 2.59 ms), rectus femoris (by an average of 19.25%, 0.68 ms) and gastrocnemius (by an average of 34.50%, 1.74 ms), (Table 3B). Finally, improvement in daily activity measured by step or arm movements count was recorded and revealed an increase by an average of 111.77%, (Fig. 2, 3 and 4; Table 3A). The Ejection Fraction (EF) and Fractional Shortening (FS) parameters values assessed by echocardiography (ECHO) were maintained and comparable with the baseline values in all patients over the entire 6-months follow-up period of observation, (Fig. 2, 3 and 4).

#### Summary of the preliminary efficacy outcomes of the first three patients enrolled to the study

##### Patient 1 (ambulatory)

Preliminary efficacy measures were assessed on the subsequent follow-up visits after systemic administration of the single dose of DEC01 therapy (Fig. 2). At the 6-month’s visit, improvements in the functional tests were recorded and revealed: 6MWT with increase of 39 m in the 6 MW distance – improvement of 9.07% (Fig. 2a; Table 3A), Timed NSAA test revealed decline in ability to stand from supine by 1.92 s and improvement in other timed test of NSAA: 10-m walk/run by 14.03% (time shorter by 0.7 s), (Fig. 2, b and c; Table 3A). Compared to the presence of the Gowers's sign at the screening visit, the patient lost Gowers's sign during assessment at 3 and 6-months post DEC01 administration. Recordings of steps count by Garmin Vívosmart 4 went from an average of 3120 ± 163 steps per day (the weeks 3–5 following the DEC01 administration when recordings started) to an average of 9084 ± 459 steps per day at 6-months post-treatment. The gains in steps count were not uniform; some days the number of steps decreased, but the overall trend revealed a steady rise confirming improvement by 191.15% (P ≤ 0.0001) at the 6-month time point (Fig. 2d; Table 3A).

Moreover, the patient maintained over the 6 months follow-up his upper limb function assessed by PUL test, including all three levels: the high-, the mid- and the distal-level of muscle function (Fig. 2, e–g; Table 3A). The skeletal muscle function assessed by standard hand grip strength measurement with dynamometer, revealed improvement in the right hand by 32.00% (from 5.0 kg at the baseline to 6.6 kg at 6-months post-transplant) and in the left hand by 40.00% (from 4.0 kg to 5.6 kg), (Fig. 2h; Table 3A).

To further analyze the restoration of skeletal muscle activity and function, electromyograms were performed. At 6 months post-transplant, electromyography of the selected muscles demonstrated improvements in the average duration of the MUP: in deltoideus by 14.52% (P ≤ 0.01), biceps brachii by 78.79% (P ≤ 0.01), rectus femoris by 40.37% (P ≤ 0.05) and gastrocnemius by 28.38% (Fig. 2, i-l; Table 3B).

The EF and FS parameters values assessed by ECHO were maintained and comparable with the baseline values over the 6-moths follow-up period (Fig. 2, m and n).

##### Patient 2 (non-ambulatory)

Assessments of the preliminary efficacy outcomes performed at 6-months follow up visit (Fig. 3) revealed significant improvement in PUL 2.0 test score from 0 points with no useful hand activity to 2 points (at 1-month visit) and 3 points (at 3- and 6-month’s visits)—with the patient regaining his ability to raise a loaded cup to the mouth (Fig. 3a; Table 3A). Additionally, patient improved in mid- and distal- PUL level score (Fig. 3b; Table 3A), with a total score increase from 20 to 23 (improvement by 15%), (Fig. 3c; Table 3A). The results of Performance of Upper Limb assessment correlated with the improvement of grip strength in both, the right hand by 22.50% (from 8.0 kg to 9.8 kg, P ≤ 0.01) and in the left hand by 26.51% (from 8.3 kg to 10.5 kg, P ≤ 0.001), (Fig. 3d; Table 3A).

At 6 months after DEC01 administration, electromyography confirmed increase in the average MUP duration in deltoideus by 90.77% (P ≤ 0.0001), in biceps brachii by 142.92% (P ≤ 0.0001), and in gastrocnemius muscle by 29.81% (P ≤ 0.0001), whereas the average MUP duration for rectus femoris was maintained at baseline level (Fig. 3, e–h; Table 3B).

Patient average arm movement counts recorded by Garmin Vivosmart 4 were steadily increasing after DEC01 treatment from 158 ± 17 daily at baseline to 277 ± 63 at 6-months post-DEC01 therapy administration revealing improvement by 75.32% (P ≤ 0.05), (Fig. 3i; Table 3A).

The EF and FS parameters values assessed by ECHO were maintained and comparable to the baseline values over the 6-month follow-up period (Fig. 3, j and k).

##### Patient 3 (ambulatory)

Assessment of preliminary efficacy outcomes performed at 6-months follow up visit (Fig. 4) revealed improvements in the functional tests when compared to the baseline values: in 6MWT by 15.73% (53 m), in the NSAA supine to stand timed test by 5.35% (time shorter by 0.4 s), while the 10 m walk/run NSAA test revealed decline by 7.03% (time longer by 0.31 s) (Fig. 4, a-c; Table 3A). Patient average step counts increased from an average of 5634 ± 363 steps per day at baseline to the 9512 ± 692 steps per day at 6 months post-transplant, confirming improvement of 68.83% (P ≤ 0.001), (Fig. 4d; Table 3A).

Moreover, PUL test confirmed improvement in the high- (83%, from 6 to 11 points) and distal-level (20%, from 10 to 12 points) and patient maintained his abilities in both, the entry and mid-level scores, resulting in the total score increase from 32 to 39 points – the increase by 21.88% compared to baseline (Fig. 4, e–g; Table 3A). Additionally, a grip strength of the right hand improved by 6.90% (from 2.9 kg at baseline to 3.2 kg at 6-months post-transplant), (Fig. 4h; Table 3A).

Electromyography confirmed increase in the average MUP duration in all tested muscles: in the deltoideus by 57.98% (P ≤ 0.001), in biceps brachii by 46.13% (P ≤ 0.0001), in the rectus femoris by 18.97% and in gastrocnemius by 41.58% (P ≤ 0.0001), (Fig. 4, i - i l; Table 3B).

The EF and FS parameters values assessed by ECHO were maintained and comparable to the baseline values over the 6-months follow-up (Fig. 4, m and n).

## Discussion

The most obvious deficiency of DMD patients is their progressive skeletal muscle weakness. As the patients get older the skeletal muscle weakness becomes more severe and the cardiopulmonary symptoms begin to appear. Cardiac disease, especially fibrosis, is now the number one cause of death for DMD patients [48]. Moreover, all patients over 18 years old will have cardiac pathology [48]. The second leading cause of death is respiratory failure, such as aspirations and infections [4, 49, 50]. Therefore, all three muscle groups must be effectively treated or, better yet, must be prevented from illness in the first place by treating the patients during earlier stages of the disease.

We have now shown the safety and preliminary efficacy data from a first-in-human study of DEC01 therapy – a novel DMD treatment that is designed to restore dystrophin expression in the muscle tissues affected by DMD [36, 37, 40–42], without eliciting an immune response [45]. Included in the data set are lack of treatment related AEs and SAEs, the absence of anti-HLA antibodies in all three patients confirms the safety and tolerability of treatment.

Moreover, the preliminary efficacy outcomes collected at 6-month post DEC01 administration showed improvement in some objective tests. The two ambulatory patients had improvements in their 6MWT and the two, timed functions of NSAA (supine to stand and 10 m walk/run test). Importantly, by 3- and 6-months post-DEC01 administration patient 1 lost the Gowers's sign, whereas patient 3 who showed Gowers’s sign at the baseline, lost the Gowers’s sign at 8-months post-transplant between the scheduled visits. Both the ambulatory and non-ambulatory patients improved or maintained the functions assessed by the PUL 2.0 test. Moreover, the non-ambulatory patient regained the ability to raise a loaded cup to the mouth, which was lost before DEC01 administration due to DMD progression. The improved muscle strength and function correlated with increase of the MUP duration assessed by electromyography in all three patients in the selected muscles of upper (deltoideus, biceps brachii) and lower (rectus femoris and gastrocnemius) extremities. These improvements correlated with the increases in the daily steps count for ambulatory patients and arm movements count for the non-ambulatory patient.

Additionally, the assessment of Ejection Fraction (EF) and Fractional Shortening (FS) by echocardiography (ECHO) demonstrated preservation of cardiac function in all patients**.**

Both the improvements in the functional tests, EMG assessment and stability of cardiac function described above are seen as a win; in such a progressive disease in untreated patients, not phenotypically progressing is desirable. This underscores that diagnosing and treating young patients is likely better, but that older non-ambulatory patients will still benefit from the DEC01 therapy. This was later demonstrated by patient 2 who, although non-ambulatory still had improvements in many metrics: PUL, average duration of MUP and daily arm movements, and did not have decreases in cardiac parameters.

The DEC01 therapy has a number of benefits beyond effectively treating the skeletal muscles and inhibiting disease progression in cardiac function. DEC01 therapy is designed to prevent triggering an immune system response. We have now shown that indeed it does not lead to generation of the immune response revealed by negative testing for anti-HLA antibodies. This confirms a major advantage of DEC01 therapy that does not require immunosuppression. Moreover, when compared with other treatments [27, 51–53], DEC01 therapy is not associated with any genetic manipulation and therefore involves no risk of off target mutations. As the approach does not use viral vectors, therefore can be readministered if needed. Another major asset of DEC01 therapy is that it is not dependent on the genetic mutation of the DMD patient, thus making DEC01 a universal therapy for all DMD patients, and other muscular dystrophy patients.

Some limitations of the study should be mentioned. This is the first preliminary report of a Pilot first-in-human study based on the outcomes from three DMD patients. Additional data from these three patients and others enrolled to the study will soon be forthcoming, including a complete report of cardiac and skeletal muscle functions. However, the presented evidence of functional improvements up to 6-months post-DEC01 administration in all three patients is encouraging considering the progressive nature of DMD.

Our preclinical studies, confirmed restoration of dystrophin expression in the target organs of heart, diaphragm and gastrocnemius muscle which correlated with reduced inflammation and fibrosis, reduced mdx muscle pathology, normalization of muscle fibers architecture and improved function confirmed by objective tests of echocardiography, plethysmography and standard functional tests up to 180 days after systemic-intraosseous DEC administration [40–42].

In a clinical scenario, assessment of dystrophin by immunoblots requires patient tissue from an invasive open biopsy under anesthesia. This exposes DMD patients to anesthesia related complications [54]. Thus, due to the safety concerns, we have not performed immunoblots for dystrophin protein detection on patient tissues before and after treatment. Furthermore, reliable results from immunoblots are difficult to obtain on such a large protein as dystrophin [55, 56]. These concerns were recently addressed at the TREAT-NMD / World Duchenne Organization Meeting, where the reliability and safety of muscle biopsy for western blot assessment were discussed [56]. Therefore, we have opted for the less invasive electromyography assessment which is an established method for evaluation of DMD progression and can be considered as the reliable and sensitive electrophysiological biomarker of restoration of dystrophic muscle activity and function in DMD patients [57–59].

Therefore, electromyograms experiments and results confirming improvements after DEC01 treatment are of particular importance. The electromyography is a minimally invasive, behavior-independent method resulting in objective and quantitative data. The duration of the MUP depends on the depolarization of many muscle fibers that constitute the given motor unit with the terminal nerve branch and that are both away from and close to the tip of the needle. It best reflects the number of functional muscle fibers and, unlike the amplitude, does not depend on the distance of the needle from the firing muscle fibers. Therefore, regenerating motor units display increasing MUP durations [60]. Thus, significant increase in MUP duration in the selected muscles of upper and lower extremities of both the ambulatory and non- ambulatory patients correlating with improvement of functional tests indicates rescue of the dystrophic muscle and regenerative potential of DEC01 therapy assessed at 6 months after systemic – intraosseous administration. Moreover, EMG was also assessed by other investigators as the objective biomarker of DMD progression, further confirming the important role of electromyography in the assessment of electrophysiological changes in the skeletal muscles of DMD patients [57].

In conclusion, this Pilot first-in-human study confirms the safety and efficacy of DEC01 treatment with absent AE and SAE over an average 12-month observation period. In addition, safety of the systemic – intraosseous DEC01 administration was confirmed in the absence of immune responses confirmed by lack of presence of anti-HLA antibodies over the entire follow-up period. Furthermore, DEC01 efficacy was confirmed by a steady improvement of standard and objective functional tests up to 6 months post-transplant. The functional improvements correlated with increased MUP values assessed by EMG in the selected muscles of the upper and lower extremities. Thus, since electromyography is an objective and minimally invasive method of assessment of muscle health and disease, therefore, we propose EMG as the reliable and sensitive electrophysiological biomarker of restoration of dystrophic muscle activity and function in DMD patients.

To the best of our knowledge this is the first study reporting the systemic- intraosseous method of myoblast administration and introducing Dystrophin Expressing Chimeric (DEC) cells, as a novel universal, cell-based therapy for all DMD patients regardless of gene mutation.

These findings are significant and relevant considering the progressive nature of the DMD disease and the absence of effective therapies.

## Data Availability

All data generated or analyzed during this study are included in this published article.
